# Effects of Paclobutrazol on Reproductive and Vegetative Traits of *Phalaenopsis* Join Grace ‘TH288-4’

**DOI:** 10.3390/plants13172385

**Published:** 2024-08-27

**Authors:** Yi-Chien Lu, Yu-Huan Chen, Ting-Hsuan Huang, Ruo-Yi Liu, Rong-Show Shen

**Affiliations:** 1 Department of Horticultural Science, National Chiayi University, No. 300, Xuefu Rd., East Dist., Chiayi City 60004, Taiwan; elsa860622@gmail.com (Y.-C.L.); chenoscar.41111@yahoo.com.tw (Y.-H.C.); love5203550@gmail.com (T.-H.H.); 2 School of Life Science, National Taiwan Normal University, 88 Ting-Chow Rd., Sec. 4, Taipei 11677, Taiwan; 3 Department of Horticulture and Landscape Architecture, National Taiwan University, Taipei 10647, Taiwan; ruoyi0109@gmail.com

**Keywords:** plant growth retardants, paclobutrazol, dwarf, stalk length, spike truncation, single-flower phalaenopsis

## Abstract

Phalaenopsis is the most popular potted plant worldwide. However, its typically long stalks often lead to increased shipping costs and risks. This study investigates the effectiveness of varying the concentration, timing, and frequency of paclobutrazol (PP333) applications on shortening the stalk of *Phalaenopsis* Join Grace ‘TH288-4’. Concurrently, it also examines the potential for producing visually appealing and single-flower potted phalaenopsis products by means of truncation. Mature phalaenopsis plants were moved to a cool room in the seventh week to induce flowering. Four experimental groups were established based on different PP333 application schedules: the control (CK) group, with reverse osmosis water application in the second week; the T2 group, with a single application in the second week; the T2T3 group, with applications in both the second and third weeks; and the T7T8 group, with applications in the seventh and eighth weeks. The PP333 concentrations used were 250, 500, 750, and 1000 mg·L^−1^, applied as foliar sprays. The results showed that the shortest stalks, measured from the base to the first flower, were observed in the T2 group treated with PP333 at 750 mg·L^−1^ and in the T2T3 group with PP333 at 500, 750, and 1000 mg·L^−1^. These treatments resulted in stalk lengths of 19.18–22.17 cm, which are 67.2–71.6% shorter than the controls. PP333 application had minimal effect on the stalk diameter, pedicel length, flower width, length, and length/width ratio. However, root diameter was thicker in plants treated with PP333 compared with the control plants. For producing single-flower phalaenopsis, a foliar spray of 750 mg·L^−1^ PP333 is recommended approximately a month before moving the plants to cooler conditions, followed by truncation, retaining only the first flower. As a result, this study establishes a PP333 treatment protocol for phalaenopsis, offering a strategy to effectively shorten the stalks.

## 1. Introduction

Phalaenopsis is a perennial flower crop that belongs to the Orchidaceae family. It is commercially produced worldwide as a flowering potted crop due to its variety of flower colors and shapes, long and regulable flowering periods, and high tolerance during storage and shipping. Furthermore, phalaenopsis has brought significant economic value to Taiwan over the past two decades. However, its typically long inflorescences often lead to increased shipping costs and risks [1]. Plants with overlength inflorescences need a bigger packing box and thus reduce container capacity. Once the plant is injured, ethylene will be produced in large amounts and then the plant will decay. Currently, market demand and breeding objectives tend to produce medium or small rather than huge phalaenopsis. A small plant is portable, easy to grow, and takes up little space.

Using plant growth retardants (PGRs) on floriculture crops can shorten the inflorescences, produce visually appealing orchids including *Phalaenopsis*, *Dendrobium*, and *Aranda*, simplify production management, reduce shipping costs, and stabilize yield. However, it usually results in the deformity of vegetative organs, such as leaves becoming thicker, shorter, and darker [2,3,4,5]. Soaking the bulb or tuber of bulbous and tuberous plants such as *Iris × hollandica*, *Iris nigricans* Dinsm, *Hippeastrum equestre*, and *Polianthes tuberosa* L. in PGRs solution can also shorten the inflorescence length. However, in some species, this results in smaller flowers, a lower flowering rate, and more days to anthesis [6,7,8,9]. The application of PGRs on *Primula forbesii*, *Chrysanthemum coronarium* L., *Lupinus varius*, and *Dianthus caryophyllus* ‘Mondriaan’ by drenching or spraying can dwarf the plants, shorten the inflorescences, prevent lodging, and make the plants compact [10,11,12,13].

These chemicals are also named antigibberellins since they control the plant height by blocking the gibberellin biosynthesis pathway, reducing the content of gibberellins in plants. Paclobutrazol (PP333, PBZ) (2RS, 3RS)-1-(4-chlorophenyl)-4,4-dimethyle-2-(1,2,4 Triazol-1-y) is one of the most commonly used PGRs and is more effective than others in managing the height of horticultural crops. PP333 represses internode elongation by affecting the isoprenoid pathway, inhibiting gibberellin synthesis. The main biochemical action is to block the reactions which lead to *ent*-Kaurenoic acid from *ent*-Kaurene [14]. For herbaceous potted flower crops, the general recommended concentration of PP333 is 30 mg·L^−1^ by spraying or 1 mg·L^−1^ by drenching [15]. As for other crops, the concentration which is the most effective varies by species and cultivars, environmental conditions, and expectation of response [16].

This study investigated the effectiveness of different application concentrations, timings, and frequencies of PP333 in shortening the stalks of phalaenopsis and produced stable, reliable, and reproducible single-flower phalaenopsis with approximately 20 cm stalks. This study also observed and determined whether PP333 application affects the development and morphology of organs or not. The goal was to examine the best PP333 application strategy, which is the most effective on the stalk length, with the slightest influence on the other organs, and the most feasible for the industry. Moreover, the aim was to figure out a mathematical model providing a reference and a protocol for academia and industry to produce single-flower potted or shortened phalaenopsis products.

## 2. Results

### 2.1. Effects of PP333 on Reproductive Traits in Phal. Join Grace ‘TH288-4’

**Figure 1:**
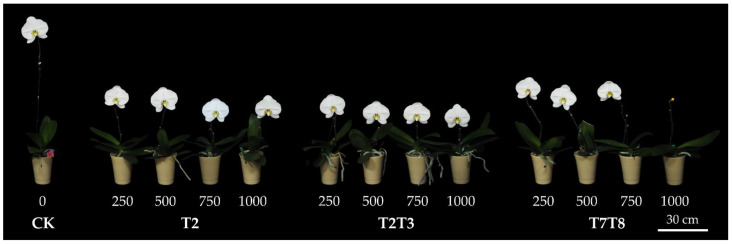
Effects of application concentration, timing, and frequency of PP333 on the plant appearance of truncated *Phal*. Join Grace ‘TH288-4’. The plant of the T7T8 group with 1000 mg·L^−1^ PP333 showed bud abortion.

All PP333 treatments inhibited the stalk length from the base to the first flower by 39.2–71.6% compared with the CK group. The stalk elongation in each PP333 treatment was significantly dwarfed compared with the CK group. The greatest inhibitory effect was observed in the T2 group with PP333 at 750 mg·L^−1^ and the T2T3 group with PP333 at 500, 750, and 1000 mg·L^−1^. Even at the same concentration, each group showed different results due to the timing and frequency of the PP333 applications. PP333 was applied to both the T2T3 and T7T8 groups twice but at different timings. The application of the T2T3 group was one month earlier than that of the T7T8 group. PP333 was applied to both the T2 and T2T3 groups before they were moved to a cool room, while it was applied to the T2T3 group twice. That was the reason why the stalk length of the T2T3 group at each PP333 concentration was shorter than that of the T2 and T7T8 groups (Table 1) (Figure 1).

**Figure 2:**
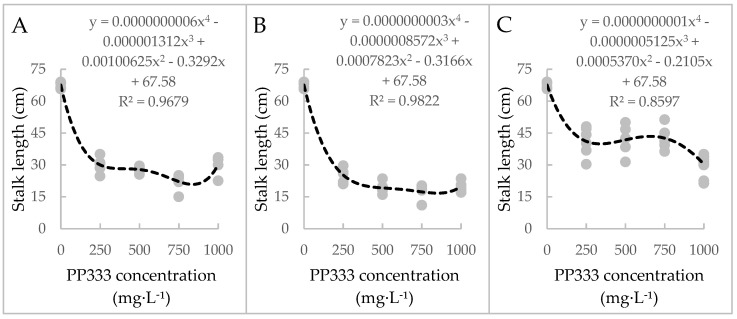
Correlation between the PP333 application concentration and the stalk length from the base to the first flower of (**A**) the CK and T2 group, (**B**) the CK and T2T3 group, and (**C**) the CK and T7T8 group in *Phal*. Join Grace ‘TH288-4’. The dashed lines are the trendlines for the effect of PP333 concentration on the stalk length from the base to the first flower estimate of persuasion.

The stalk length from the base to the first flower got shorter when the concentration of PP333 was higher. Nevertheless, in the T2 group, the stalk length treated with PP333 at 1000 mg·L^−1^ was higher than 500 and 750 mg·L^−1^ and was the same as 250 mg·L^−1^. As for the T2T3 group, when the concentration was above 500 mg·L^−1^, the stalk length of each treatment was statistically identical. The stalk length of each treatment of the T7T8 group was higher than the other groups (Table 1) (Figure 1 and Figure 2). Figure 2 showed correlation between the PP333 concentration and the stalk length from the base to the first flower, and each dot represented data of a plant. Three groups showed a similar tendency that the stalk length got shorter when the concentration of PP333 was higher. However, based on different groups, the trendlines are slightly different. The R-squared value of trendline of the CK and T2T3 group was higher, representing smaller differences between the observed data and the fitted values than the others (Figure 2B).

**Figure 3:**
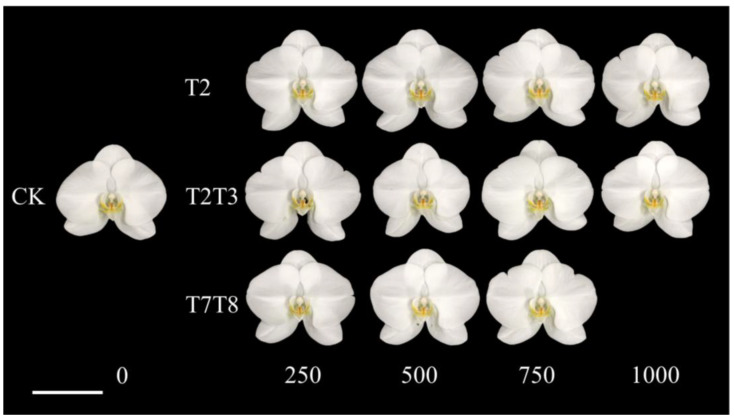
Effects of application concentration, timing, and frequency of PP333 on the flower appearance of *Phal*. Join Grace ‘TH288-4’. None of the first flowers of the T7T8 group with 1000 mg·L^−1^ PP333 were in anthesis.

**Figure 4:**
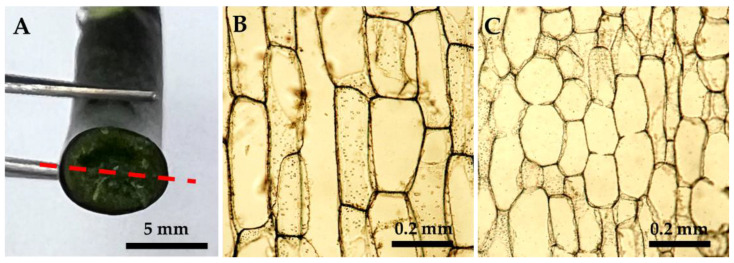
Effects of application of PP333 on the stalk sagittal sections of *Phal*. Join Grace ‘TH288-4’. (**A**) Schematic diagram of the sagittal sections of the stalk; the red dashed line is the cut section; (**B**) the stalk sagittal sections of the CK group; and (**C**) the stalk sagittal sections of the T2T3 group with 1000 mg·L^−1^ PP333.

There was no deformity on the flowers in all treatments, and the appearance, such as flower shape, color, and structures, was not affected by the PP333 treatments (Figure 3). Regarding the stalk diameter, only treatments with high concentrations of PP333 applied twice had a minor effect. The stalk sagittal sections showed that the cells of the CK group (Figure 4B) were more cramped and longer than those of the T2T3 group treated with 1000 mg·L^−1^ PP333 (Figure 4C). There was no effect on other productive traits, including flower width, length, length/width ratio, and pedicel length (Table 1). Additionally, the appearance of the stalk and flower were not affected by the application of PP333 (Figure 1 and Figure 3).

### 2.2. Effects of PP333 on Vegetative Traits in Phal. Join Grace ‘TH288-4’

**Figure 5:**
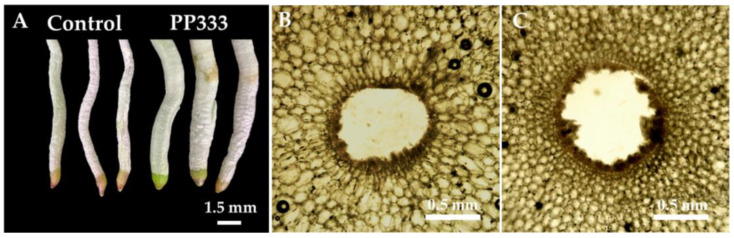
Effects of application of PP333 on the root of *Phal*. Join Grace ‘TH288-4’. (**A**) The new roots randomly cut from the CK group and the PP333 treatment plants. (**B**) The root transverse sections of CK group. (**C**) The root transverse sections of the T2T3 group with 1000 mg·L^−1^.

When the plants were treated with PP333, the new roots turned out to be thicker than those of the CK group. The root diameter of the CK group was 6.72 mm, while those treated with PP333 at any concentrations were 9.38–10.42 mm (Table 2). Randomly cut new roots from the CK group and the PP333 treatment plants indicated that the PP333 treatments resulted in thicker new roots (Figure 5A). The cells of new roots cut from the T2T3 group treated with 1000 mg·L^−1^ PP333 were smaller, denser, and more compact than those of the CK group (Figure 5B,C).

Some vegetative traits, including the leaf length, leaf width, and leaf shape, were slightly influenced by the PP333 treatment. The leaf length of the CK group was shorter than the others and the leaf width was wider. The leaf length/ width ratio of those treated with PP333 mostly were higher, referring that the leaves were slenderer than those of the CK group. As for the leaf span, leaf number, and leaf NDVI value, these were affected trivially by PP333 application. Only few treatments showed statistically significant difference with the others (Table 2). There was no abnormality that was caused by applying PP333 on the whole plant excluding roots (Figure 1).

### 2.3. Truncation in Phal. Join Grace ‘TH288-4’

The spike truncation treatment did not influence the stalk, bud, and flower development and blooming. After the spikes were removed, the wound sections healed. The stalk stopped growing and the first flower bud remained developing until blooming.

## 3. Discussion

### 3.1. Effects of PP333 on Reproductive Traits in Phal. Join Grace ‘TH288-4’

After foliar spraying of PP333, all treatments with different concentrations of PP333 significantly dwarfed the stalk length compared to the CK group (Table 1) (Figure 1 and Figure 2). This result is consistent with plenty of research [1,2,3,6,8,11,13,17,18,19,20]. After the phalaenopsis (*Phal*. *amabilis* Blume × *Phal*. Mount Kaala ‘Elegance’) was moved to the cool room for four weeks, the plants were sprayed with 500 mg·L^−1^ PP333, and the stalk length from the base to the first flower was shortened by 13.6% compared to the controls [1]. Spraying 1.0 mL·L^−1^ PP333 on the leaves of *Phal*. Nobby’s Amy twice can repress the stalk length from the base to the first flower by 59.8% compared to the controls [2]. Irrigating *Phal*. Sogo Yukidian ‘V3’ with 200 mL of 5, 10, 20, or 40 mg·L^−1^ PP333 per pot or spraying with 13 mL of 200 or 400 mg·L^−1^ PP333 per pot can shorten the inflorescence length by 21.2–38.8% compared to the controls [3].

In terms of the application timing of PP333, earlier application showed a better dwarfing effect. The results of the T2T3 and T7T8 groups showed that the stalk length of the T2T3 group was shorter than that of the T7T8 group. For example, the stalk length of the T2T3 group treated with 250 mg·L^−1^ PP333 was 25.27 cm and was 62.6% shorter than that of the CK group, while the stalk length of the T7T8 group was 41.08 cm and was only 39.2% shorter. Furthermore, in higher concentrations of PP333, the differences between the T2T3 and T7T8 group were greater (Table 1) (Figure 1). This result aligns with the findings of two studies [1,20]. The later the PGRs is applied before the stalk grows to 10 cm, the weaker its ability to inhibit the stalk length. Applying PP333 at 250 mg·L^−1^ before *Phal. amabilis* Blume × *Phal*. Mount Kaala ‘Elegance’ stalk emergence results in the shortest stalk length of 22.1 cm, which is 54.6% shorter than the controls [1]. At the same PP333 concentration, the T2T3 group in this study had better dwarfing stalk effects, likely due to being sprayed twice and with earlier application timing.

Regarding the application frequency of PP333, the dwarfing effect was better with two applications. The data from the T2 and T2T3 groups showed that the stalk length of the T2T3 group was shorter than that of the T2 group. In each concentration, the stalk length of the T2T3 group was shorter than that of the T2 group (Table 1) (Figure 1). This phenomenon was consistent with research that also treated *Phal*. with PP333. Applying PP333 1.0 mL·L^−1^ twice resulted in the stalk length being 59.8% shorter than the controls, while a single application resulted in the stalk length being only 17.1% shorter than the controls [2].

The stalk length decreased as the concentration of PP333 increased, and this phenomenon was consistent with previous research [1,2,3]. However, the stalk length was not significantly different when the concentration exceeded 500 mg·L^−1^ PP333 in the T2T3 group. In the T2 group, the stalk treated with 1000 mg·L^−1^ PP333 was not the shortest. It was speculated that the unusual phenomena, such as inconsistency of the correlation between the stalk length and the concentration of PP333 among three groups, the bending stalk of the T2 and T7T8 group, and the bud abortion of the T7T8 group applied with 1000 mg·L^−1^ PP333, may result from relatively small sample sizes of each group, different location causing uneven light intensity, and leaking raindrops. Since the cool room is cramped, the sample size of each treatment was only six plants and may lead to the outliers impacting the statistical results. The cool room was equipped with an air conditioner and circulator, but they were hung from the ceiling and inevitably shaded the plants under them, resulting in bending stalks due to phototropism. Sometimes, a little of the raindrops dripping on the plant caused the wound sections to be infected, then resulted in bud abortion, wither, and etiolation.

The trendline offers a reference to determine which concentration is better and suitable for each situation. Based on different groups, the trendlines are slightly different (Figure 2). The R-squared value of Figure 2C, which analyzed the CK and T7T8 group, was the lowest, suggesting that the trendline was less reliable than the others (Figure 2C). Because the data of the T7T8 group were more scattered, the late application time may have caused each plant to react discrepantly to PP333.

The ideal height of a potted plant is 2–2.6 times the pot diameter [21]. The plant material used in this study is *Phal*. Join Grace ‘TH288-4’ cultivated in 10.5 cm pots, so the ideal plant height is approximately 21.6–28.1 cm. Since the aim of this study was to produce an orchid with only one flower, unlike most previous studies on multiple flowers, the ideal plant height was lower, about 20 cm. According to the results of this test, in order to achieve the ideal single-flower phalaenopsis, 19.18–22.17 cm was the most appropriate stalk length below the first flower, and their PP333 treatments were the T2 group with 750 mg·L^−1^ PP333 and the T2T3 group with 500, 750, and 1000 mg·L^−1^ PP333. Thus, the minimum effect dose of PP333 of the T2T3 group was only 500 mg·L^−1^, yet the T2 group was 750 mg·L^−1^. Most managers of the phalaenopsis company usually tend to choose the most economical, time-saving, and efficient strategy. Treatment with 500 mg·L^−1^ PP333 costs one more application and a higher PP333 dose, causing higher personnel and material costs. Thus, applying only once with 750 mg·L^−1^ PP333, rather than twice with 500 mg·L^−1^ PP333, was recommended as the most appropriate treatment.

The average stalk diameter of the CK group was 9.80 mm, with no significant difference from that of the T2 group. The stalk of the T2T3 and T7T8 groups were significantly thicker than those of the CK and T2 group (Table 2). This result was similar to one study and may be caused by application frequency [3]. The PP333 content in plant applied twice was extremely likely higher than that of one application, and thus causing the thicker stalk. There were no significant differences in pedicel length, flower width and length, flower length/width ratio, and flower shape between different application concentrations, timings, and frequencies of PP333 (Table 1) (Figure 1). This result is consistent with [1,2,3].

### 3.2. Effects of PP333 on Vegetative Traits in Phal. Join Grace ‘TH288-4’

In this study, the root diameter of plants treated with PP333 was significantly thicker than that of the CK group. This phenomenon is consistent with many previous studies [1,2,3,22].

There was only a slight difference in leaf shape, which was not severely deformed and rounded as in previous studies (Table 2) (Figure 1). It was speculated that this was mainly due to leaves of the cultivar, Join Grace ‘TH288-4’, being less affected by PP333. Also, the light quality is one of the factor that effects the leaf shape. The roof of this study and the others may be made of different materials.

There was no significant difference in leaf span between the CK group and treatment groups, except for the T2T3 group treated with 1000 mg·L^−1^ PP333, which showed the minimum value of 37.90 cm. The number of leaves in the CK group was slightly higher, due to the removal of diseased and old leaves resulted in slight differences among the treatments. There was little difference in leaf NDVI values among treatments. It is speculated that these differences were caused by the limited space in the greenhouse, which inevitably affected leaf expansion and overlap, but not by the effectiveness of PP333 (Table 2).

### 3.3. Truncation in Phal. Join Grace ‘TH288-4’

Only the plant of the treatment with 1000 mg·L^−1^ PP333 of the T7T8 group failed to bare a bud and bloom. This may due to the dripping water from the roof at the cultivation region of the T7T8 group rather than the truncation.

## 4. Materials and Methods

### 4.1. Plant Materials and Environment

Mature *Phal*. Join Grace ‘TH288-4’ plants, potted in 10.8 cm plastic pots with moss, produced by Join Orchids (Xiaying Dist., Tainan City, Taiwan), were used for this experiment. Each plant had 5–7 leaves spanning 41.93 cm on average, and the data are shown in Table 3. The experiment was conducted in C1 or C3 rooms at the Horticultural Technology Center, National Chiayi University. The roof, made of polyethylene, was equipped with a shading net. When the light intensity reached 463 µmol·m^−2^·s^−1^, the shading net automatically covered to reduce light by 60% to prevent sunburn. The greenhouses were also equipped with air conditioner, circulator, atomizers, and supplemental lighting. The relative humidity was controlled as 60–80%. The air conditioner was also capable of dehumidification function. While the relative humidity was below 60%, the humidifier sprayed reverse osmosis water to promote moisture.

### 4.2. Method of the PP333 Treatments

According to the application timing and frequency of PP333 (Chia-Tai Co. Ltd., Chiayi City, Taiwan), four groups were set up: control (CK), T2, T2T3, and T7T8. The CK group applied at the 2nd week with reverse osmosis water; the T2 group had only one application at the 2nd week; the T2T3 group had two applications at both the 2nd and 3rd weeks; and the T7T8 group also had two applications at the 7th and 8th weeks (Table 4).

All treatment groups received PP333 at concentrations of 250, 500, 750, and 1000 mg·L^−1^ by foliar spray. Each treatment contained six plants, the CK group with six plants and the T2, T2T3, and T7T8 with twenty-four plants, respectively. All solutions contained 0.05% Tween 20 (Choneye Pure Chemicals, Chiayi City, Taiwan), which was mixed well with reverse osmosis water in handheld spray bottles and then sprayed on the stems and upper and lower epidermis of the leaves. We prepared 50 mL PP333 solution at different concentrations for each treatment, and each plant was sprayed till there was water droplets on the surface of the leaves. Thus, the PP333 solutions were applied at an average rate of 12 mL per plant.

**Figure 6:**
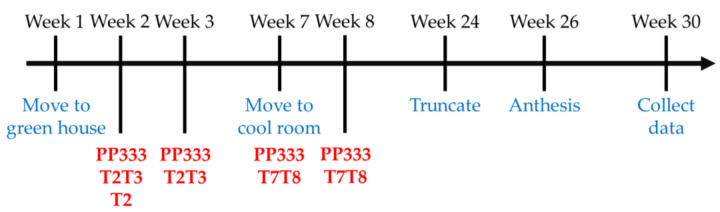
Flow chart of the test of application concentration, timing, and frequency of PP333 on the reproductive and vegetative traits in *Phal*. Join Grace ‘TH288-4’.

All plants were moved to the C3 room set as 30/25 °C on 5 October 2022 (week 1) and then were transferred to the C1 room set to 20/18 °C to induce flowering on 9 November 2022 (week 7). On 8 March 2023 (week 24), the first flower buds of a few plants were 1–1.5 cm long, so their spikes were truncated with autoclaved scalpels. We cut the stalk on the position right beside the connection of the pedicel of the first bud and the stalk, then removed the spike. Only the first bud and the stalk from the base to the first flower remained and kept on cultivating till anthesis. The truncation of all plants lasted for approximately a week. After two weeks, the phalaenopsis started to bloom. The earliest anthesis was the plant of the T2 group applied with 1000 mg·L^−1^ PP333, taking 142 days from flowering induction (9 November 2022). The progress is shown in Figure 6.

During the experiment, irrigation occurred once every two weeks, completing four cycles. The first and second irrigations used tap water with 1 mg·L^−1^ Jack’s Professional^®^ (20-20-20 General Purpose, J.R. Peters Inc., Allentown, PA, USA), the third irrigation with 0.5 mg·L^−1^ Jack’s Professional^®^, and the fourth irrigation used only tap water. The first, second, and third cycles were irrigated with 200 mL per plant and the fourth with 400 mL per plant.

### 4.3. Data Collection

Data collection began when the first flowers fully expanded on 19 April 2023. Reproductive traits measured included the stalk length, stalk diameter, pedicel length, flower width, and flower length/width ratio. The stalk length was measured from the base to the position of the connection of the pedicel of the first flower with a tape measure. The stalk diameter was measured using the position the between second and third node of the stalk with a digital caliper (500-196-20 Absolute AOS Digimatic, Mitutoyo Corporation, Kawasaki, Japan). The pedicel length was measured from the connection of the pedicel and the flower to the connection of the pedicel and the stalk with a tape measure. The flower width was measured as the maximum width in the horizontal direction of the fully expanded first flower, and the flower length was the maximum length in the vertical direction. The flower width/length ratio was the flower width divided by the flower length.

Vegetative traits measured included the root diameter, leaf length, leaf width, leaf length/width ratio, leaf span, leaf number, and leaf NDVI value. The root diameter was measured as the diameter of the root on the boundary of the green root tip and silver mature root with a digital caliper. The leaf length measured the longest length of the youngest mature leaf with a tape measure along the leaf midrib. The leaf width measured the widest of the youngest mature leaf with a tape measure. The leaf length/width ratio was the leaf length divided by the leaf width. The leaf span measured the longest distance between two leaf apexes with a tape measure. The leaf number was the amount of healthy and mature leaves. If the leaf was half shorter than a mature one, it was recorded as 0.5; if the leaf length was longer than half of a mature one, it was recorded as 1. The leaf NDVI value measured the youngest mature leaf with a PlantPen NDVI 300 (Photon Systems Instruments, Průmyslová 470, 664 24 Drásov, Czech Republic).

### 4.4. Statistics and Plotting

Statistical analysis was performed using Costat 6.4 (Cohort Software, Monterey, CA, USA) to conduct analysis of variance and calculate the least significant difference to determine if the quantitative traits of each cultivar exhibited significant differences at *p* < 0.05. The graphs and trendlines were created with Microsoft^®^ Professional Plus 2016 Excel to generate scatter charts, trendlines, and R-squared values.

## 5. Conclusions

**Figure 7:**
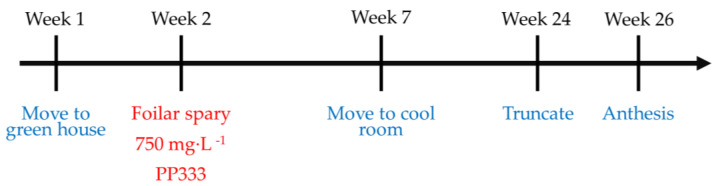
The protocol of the PP333 and spike-truncated treatments on *Phal*. Join Grace ‘TH288-4’.

The optimal stalk length for single-flower potted phalaenopsis in this experiment was determined to be 19.2–22.2 cm to achieve the ideal dwarfing effect. According to the results, the T2T3 group required a concentration of 500 mg·L^−1^ PP333 and the T2 group required 750 mg·L^−1^. Generally speaking, the industry usually takes the advice which costs less and has a simple and time-saving operation. As a result, to quickly and stably produce single-flower phalaenopsis products, the recommended treatment procedures of PP333 are as follows: at week 1, acquire phalaenopsis and place the plants in a greenhouse set at 30/25 °C; at week 2, apply 750 mg·L^−1^ PP333 only once by foliar spray; at week 7, move the plants to the cool room set at 20/18 °C; at week 24, when the first flower bud is 1.5 cm, truncate the spike and the first flower will anthesis after two weeks (Figure 7). Those who try to determine which concentration of PP333 is suitable for *Phal.* could take the trendlines of Figure 2 as the reference. Based on the timing and frequency, a trendline should be chosen to calculate the needed concentration of PP333.

## Figures and Tables

**Table 1:** Effects of application concentration, timing, and frequency of PP333 on the reproductive growth traits of *Phal.* Join Grace ‘TH288-4’.

Group	PP333 Concentration (mg·L^−1^)	Stalk Length (cm)		Stalk Diameter (mm)		Pedicel Length (cm)		Flower Width (cm)		Flower Length (cm)		Flower Length/Width Ratio	
CK	0	67.58	a	9.80	efg	5.50	a	13.33	bcd	12.27	bcd	0.93	ab
T2	250	30.00	de	9.83	efg	4.48	b	14.68	abc	13.80	a	0.94	ab
	500	27.84	defg	9.80	efg	4.48	b	12.96	cd	13.00	abcd	1.00	a
	750	22.17	fghi	10.16	efg	4.68	ab	13.26	cd	12.88	abcd	0.97	ab
	1000	30.15	de	9.50	g	5.04	ab	13.92	bcd	12.50	bcd	0.90	ab
T2T3	250	25.27	efg	10.50	defg	4.65	ab	13.72	bcd	13.33	abcd	0.97	ab
	500	19.18	hi	10.83	bcde	4.70	ab	13.13	cd	13.33	abcd	1.03	a
	750	19.43	hi	11.50	abcd	4.08	b	14.68	abc	12.56	abcd	0.85	b
	1000	19.33	hi	11.00	abcde	4.67	ab	11.67	d	11.93	cd	0.84	b
T7T8	250	41.08	b	10.67	cdef	4.75	ab	13.48	bcd	13.00	abcd	0.97	ab
	500	38.90	bc	10.17	efg	5.08	ab	13.80	bcd	13.76	ab	1.00	a
	750	42.60	b	10.40	defg	4.57	ab	13.87	bcd	12.77	abcd	0.92	ab
	1000	32.28	cd	11.00	abcde	-		-		-		-	
Significance													
Group (G)		***		***		NS		NS		NS		NS	
Concentration (C)		***		NS		NS		NS		NS		NS	
G × C		***		NS		NS		NS		NS		NS	

a–i Mean separation within columns followed by the different letter(s) are significantly different at *p* < 0.05 level by LSD. NS or ***, nonsignificant or significant at *p* < 0.001, respectively. - All the first flowers of the T7T8 group with 1000 mg·L^−1^ PP333 failed to anthesis and data could not be collected.

**Table 2:** Effects of application concentration, timing, and frequency of PP333 on the vegetative growth traits in *Phal.* Join Grace ‘TH288-4’.

Group	PP333 Concentration (mg·L^−1^)	Root Diameter (mm)		Leaf Length (cm)		Leaf Width (cm)		Leaf Length/Width Ratio		Leaf Span (cm)		Leaf Number		Leaf NDVI Value	
CK	0	6.72	d	22.58	e	8.80	a	2.58	d	45.10	def	6.00	ab	0.49	abcd
T2	250	9.57	abc	24.73	bcde	8.05	cdef	3.08	abc	44.35	ef	5.58	abcd	0.47	cde
	500	9.84	ab	25.50	abc	8.18	bcde	3.12	abc	49.24	abcd	6.50	a	0.47	de
	750	10.42	a	26.22	ab	8.18	bcde	3.21	ab	47.12	bcde	6.00	ab	0.48	bcde
	1000	9.32	abc	26.45	a	8.35	abc	3.17	ab	50.70	ab	6.00	ab	0.48	bcde
T2T3	250	10.38	a	25.10	abcd	8.13	bcde	3.09	abc	47.08	bcde	4.42	e	0.49	abcd
	500	10.10	a	23.10	de	8.18	bcde	2.83	cd	43.67	f	5.25	bcde	0.49	abcd
	750	9.67	abc	24.95	abcd	7.98	cdef	3.13	abc	48.90	abcd	4.75	de	0.52	a
	1000	9.96	ab	25.04	abcd	7.74	ef	3.23	a	37.90	g	4.80	de	0.49	abcd
T7T8	250	10.12	a	25.07	abcd	8.23	bcd	3.05	abc	48.32	abcd	6.00	ab	0.48	bcde
	500	10.32	a	25.18	abcd	8.00	cdef	3.16	ab	45.08	def	5.42	bcde	0.47	de
	750	10.24	a	25.32	abcd	7.92	cdef	3.20	ab	48.46	abcd	5.00	bcde	0.48	bcde
	1000	9.38	abc	24.77	bcde	8.20	bcde	3.03	abc	45.92	cdef	4.90	cde	0.47	de
Significance															
Group (G)		NS		NS		NS		NS		*		***		***	
Concentration (C)		NS		NS		NS		NS		NS		NS		NS	
G × C		NS		NS		NS		NS		**		NS		NS	

a–f Mean separation within columns followed by the different letter(s) are significantly different at *p* < 0.05 level by LSD. NS, *, **, and *** are nonsignificant or significant at *p* < 0.05, 0.01, and 0.001, respectively.

**Table 3:** Leaf traits of *Phal*. Join Grace ‘TH288-4’ before the PP333 treatments.

Length (cm)	Width (cm)	Length/Width Ratio	Span (cm)	Numbers
25.75	8.52	3.20	41.93	5.76

**Table 4:** The experimental details of the application of PP333 in four groups.

Group	The Application of PP333		
	Concentration (mg·L^−1^)	Timing (Week (Date))	Frequency (Times)
CK	0	Week 2 (12 October 2022)	1
T2	250, 500, 750, 1000	Week 2 (12 October 2022)	1
T2T3	250, 500, 750, 1000	Week 2 and 3 (12 and 19 October 2022)	1 + 1
T7T8	250, 500, 750, 1000	Week 7 and 8 (9 and 16 November 2022)	1 + 1

## Data Availability

Data is contained within the article.
